# Does Ménière's Disease in the Elderly Present Some Peculiar Features?

**DOI:** 10.1155/2012/421596

**Published:** 2012-01-17

**Authors:** R. Teggi, A. Meli, M. Trimarchi, F. LiraLuce, M. Bussi

**Affiliations:** ^1^ENT Department, San Raffaele Scientific Institute, Vita-Salute San Raffaele University, Milan, Italy; ^2^San Raffaele Hospital, Vita-Salute San Raffaele University, Olgettina 60, 20132 Milan, Italy

## Abstract

*Object*. Aim of our study was to establish some peculiar features of Ménière's Disease (MD) in a group of elderly MD patients, in which the first vertigo spell happened when over 65 years old. *Material and Methods*. We analyzed a group of 73 younger than 65-years-old and a group of 30 elderly MD patients. All patients underwent a neurotological evaluation, an anamnestic evaluation including a lifetime history of migraine, and blood withdrawal for autoantibody screening. *Results*. Some differences were found between elderly and younger MD patients. Elderly MD patients presented a higher prevalence of Tumarkin attacks and a lower prevalence of lifetime history of migraine; moreover, they presented a faster develop of hearing loss and vertigo spells than a subgroup of 32 younger patients matched for the duration of illness. *Conclusions*. Some clinical features of MD in elderly have been pointed out. Particularly, the lower rate of migrainous history and positivity for autoantibodies often associated with MD, in our opinion, support the hypothesis of a vascular disorder acting as a predisposing factor for MD in elderly.

## 1. Introduction

Ménière's Disease (MD) is an inner ear disorder, characterized by recurrent episodes of rotational vertigo, coupled with fluctuating hearing loss and tinnitus. According to The American Academy of Otolaryngology-Head and Neck Surgery Foundation (AAO-HNSF), the diagnostic criteria for definite MD are the presence of two or more episodes of vertigo of at least 30 min with hearing loss plus tinnitus and/or aural fullness [1].

The prevalence of MD is 190 cases per 100000, and this value increases in the elderly [2].

The commonly accepted pathogenesis of MD is a raised endolymphatic pressure (hydrops), although a direct relationship between MD and endolymphatic hydrops is unproven [3, 4]. In the last few decades, several studies have analyzed possible etiological mechanisms and correlations with other diseases [5–14]. Epidemiological studies support the possibility of a correlation between MD and migraine. MD patients present a prevalence of migraine between 43% and 56%, which is significantly higher than the estimated 10% in the normal population [10, 11].

Migraine is considered per se a causal factor of recurrent vestibular symptoms, including both true rotational vertigo and subjective vertigo [5–15]; there is clinical evidence that migraine may damage the inner ear causing permanent hearing loss or vestibular deficit [16], and in children a fluctuating hearing loss has been considered as a migraine equivalent [17]. It has been supposed that the migrainous vasospasm may induce damage in the inner ear, acting as a disrupting factor for a secondary MD [9].

Since higher levels of circulating immunocomplexes and elevated autoantibody titers have been found in MD patients, the pathophysiology of MD has been supposed to be linked to an autoimmune disorder [18, 19]. An association with the PTPN*22* T allele, which has been described in other autoimmune diseases, has been reported in bilateral MD; these data support the idea of an inflammatory mechanism common to different autoimmune diseases [20].

Unilateral MD patients have a higher level of antiphospholipid antibodies than the normal population. The antiphospholipid antibodies, anticardiolipin, *β*_2_ glycoprotein 1 autoantibodies, and Lupus-Like anticoagulant are correlated with thrombotic syndrome. The antiphospholipid antibodies could mediate vascular diseases by a thrombotic mechanism [19].

Since 10% of cases present a familiar distribution, the genetics of MD has been studied [21–23], considering both possibilities of a single gene mutation provoking the syndrome (COCH gene encoding cochlin and chromosome 12p13.3 mutation) [24, 25] and acting as a predisposing factor (HLA B-27, Antiquitin) [26, 27]. Anticipation in familial MD has been described, consisting of an earlier age of onset in successive generations and more severe clinical manifestations. The genetic model may be linked to a trinucleotide repeat disorder; this mechanism is similar to other neurological diseases such as spinocerebellar atrophy 6 and myotonic dystrophy in which a channelopathy is supposed [14].

New studies on Ménière's disease (MD) in elderly patients may help bring about a prolongation of lifespan in the last decades. An earlier study on Japanese MD subjects reported a peak of onset around the fifth decade for men and in the fourth decade for women [28], with a 10-year shift from previously reported data. The neurotological evaluation of elderly MD patients found vestibular clinical features in the elderly similar to young and middle-aged MD patients except for a higher incidence of oculomotor system alterations [28]. A higher prevalence of MD in patients over 65 years has also been reported in a European population, both for a reactivation of MD and for a “de novo” MD. In elderly MD patients, a high incidence in women and of “drop attacks” has been described [29], and Tumarkin episodes may cause sudden falls. The Tumarkin attacks [30] are caused by an acute stimulation of an otolith organ in patients with MD or delayed endolymphatic hydrops; in some cases, differential diagnosis with drop attack is a puzzling dilemma [31].

The aim of our study was to establish some possible peculiar characteristics of MD in a group of elderly MD patients compared with younger MD patients.

## 2. Materials and Methods

### 2.1. Subjects

We studied 103 definite MD patients consecutively recruited at the outpatient facilities of the Vestibular Disorders Ambulatory at San Raffaele Hospital in Milan, from January 2006 until April 2011. The diagnosis of definite MD was established according to AAO-HNSF criteria (American Academy of Otolaryngology, 1995).

Patients were divided into two groups in relation to age of onset of MD. A group of 73 younger MD patients: 41 were females and 32 were males, mean age was  50.1 ± 12  years, and age of onset of MD was  39 ± 9.7  years. Two subjects presented bilateral MD. Delayed hydrops were excluded.

Between these patients, a subgroup of 32 subjects presented a lower than 6 years history of vertigo (3.4 ± 1.8 years). Mean age was 39.6 ± 10.3. Nineteen were females.

The second group was composed of 30 elderly MD patients: 13 were females and 17 males and mean age was  72 ± 4.3  years. All of them presented a lower than 6 years history of attacks and presented unilateral MD. Exclusion criterion for this second group was a lifetime history of previous vertigo episodes before 65 years of age. First vertigo attack happened 2.7 ± 2.3 years before the evaluation.

All patients underwent a full neurotological examination, including audiometric test at 1 db precision, analysis of spontaneous, and evoked nystagmus with video-oculography (VOG 25, Interacoustics). Anamnesis of Tumarkin attacks and familiar history of vertigo (till grandparents) were assessed by a senior neurotologist. All of them performed a Central Nervous System MRI.

**Figure 1:**
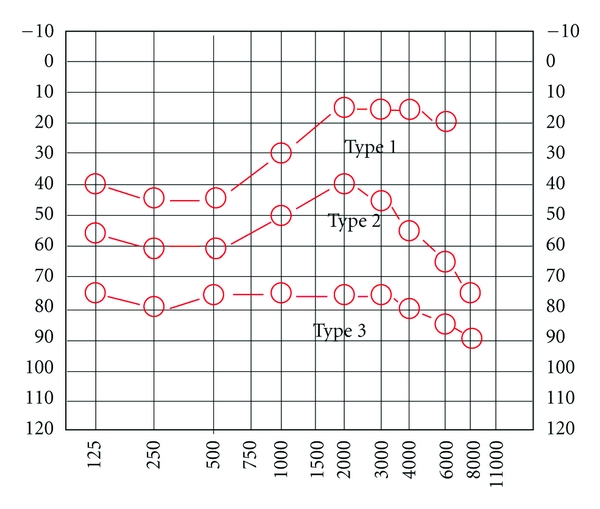
The 3 different types of audiometric exams; on *x*-axis are the represented frequencies, on *y*-axis, hearing loss in decibels (db).

Morfology of audiometric exam was divided in three groups (Figure 1).

1. Type 1: low frequencies sensorineural hearing loss with normal hearing threshold on high frequencies.
2. Type 2: reduced hearing threshold at all frequencies, increased at low and high frequencies (“peaked curve”).
3. Type 3: reduced hearing at all frequencies with a threshold below 60 db (“flatcurve”).

A lifetime history of migraine was established by a senior neurologist, according to the International Headache Society (IHS) criteria (Headache Classification Sub-Committee of the International Headache Society, 2004) [32].

An autoantibody screening (anti nucleus, mitochondrial, smooth muscle, thyroid, antiphospholipid autoantibodies, and Rheumatoid Factor) was assessed.

The study was approved by our Ethics Committee and patients signed informed consent.

### 2.2. Statistical Analysis

Continuously distributed variables were described by the mean and standard deviation (SD); categorical variables were described by frequencies and percentages. The significance of any difference between groups was evaluated by *t-*test for independent samples. A multiple linear regression analysis was performed in order to investigate the correlation between variables.

## 3. Results

Results are presented in Table 1. The elderly MD patients presented a lower rate of lifetime history of migraine (*P* = 0.04) and positive autoantibody screening (*P* = 0.04) than younger MD subjects. Only 1 patient with migraine presented positive autoantibodies.

At the time of examination, 12 of 20 (60%) elderly MD subjects presented a sensorineural hearing loss at all frequencies with a “flat” audiogram, while 8 (40%) had a peak at central frequencies. The elderly MD patients showed a higher prevalence of Tumarkin attacks (*P* < 0.01) and lower for lifetime history of migraine (*P* = 0.03) and positive autoantibodies (*P* = 0.02). Moreover, they presented a higher rate of vertigo spells in the 6 months before evaluation. Ten of 30 (33.3%) elderly MD patients presented microischemic lesions at MRI compared with 10 of 73 (13.7%) in the younger group (*χ* ^2^ = 5.2, *P* = 0.02); nine of the 10 subjects in the last group were migraineurs.

Hearing loss (mean value for frequencies between 250 and 2000 Hz) at diagnosis was 64.6 ± 8.8 db in elderly and 53.8 ± 13.8 in younger MD subjects (*P* = 0.001), while the unaffected ear (the two subjects with bilateral MD were not included) was respectively, 18.2 ± 4.5 and 16.3 ± 3.3 (*P* = 0.04).

In the whole group of 103 subjects, a correlation has been established between Tumarkin attacks and lifetime history of migraine (*P* = 0.05) and between positivity of at least one of the autoantibodies and hearing loss (*P* = 0.05).

In order to demonstrate a possible faster evolution of MD in elderly, we compared the results of hearing loss in affected and unaffected ear and the number of vertigo spells in the last 6 months in the group of elderly and in the subgroup of younger MD subjects with a shorter than 6 years history of vertigo attacks. Results and statistics are summarized in the Table 2.

In the group of elderly MD subjects 24, (80%) presented a type 3 audiogram and 6 (20%) a type 2 audiogram; while between the 32 younger MD subjects, 12 (37%) presented a type 3 audiogram, 14 (44%) a type 2, and 6 (19%) a type 1 audiogram (*χ* ^2^ = 13.1, *P* = 0.001).

## 4. Discussion

In our sample of 103 MD subjects, 30 (29%) presented onset of symptoms when over 65 years of age. Our data are in the range of other previous studies [29, 33]. In the sample of elderly MD patients, males were more represented (17 of 30, 56%), the opposite than in the younger sample 32 of 73 (44%), although result has no statistical evidence (*P* = 0.2).

Elderly subjects presented a more “aggressive” evolution of MD; these subjects referred a higher rate of vertigo spells and had a faster evolution toward a “flat” audiometric threshold (with higher values of hearing loss in the affected side) compared with the subgroup of 32 younger MD subjects matched for years since the onset of first symptom. It should be noted that the unaffected ear in elderly presented a lower hearing threshold than younger subjects, but the mean difference was only 2.1 db. As far as we know, no previous data have been published on a possible faster evolution of MD in elderly.

Elderly patients presented a higher rate of Tumarkin attacks, and on the topic our results confirm previously published works; a possible explanation may be linked to a lower compliance of otolithic structures to hydrops in elderly “de novo” MD patients [29, 34]. Alternatively, Tumarkin attacks may be linked to brief periods of ischemia or vasospasm of the anterior vestibular artery, which provides blood to the utricle, vestibular ganglion, and posterior semicircular canal. This branch is supposed to be more fragile than the posterior vestibular artery since the latter probably has a major number of intraosseous collaterals [35]. If so, the pathophysiology of Tumarkin should not differ significantly from that of paroxysmal positional vertigo, whose prevalence is significantly increased in the elderly.

The etiology of MD is at present a puzzling dilemma, and it seems probable that it consists of different pathologic conditions leading to the same cluster of symptoms. All our elderly subjects fulfill the diagnostic criteria for definite MD, but in the two groups, some clinical differences regarding possible “predisposing factors” may be noted, underlining the polymorphic nature of MD.

As previously discussed, epidemiological evidence has been found regarding an association between MD and migraine or autoimmune disorders; moreover, a genetic predisposition may be supposed, since around 10% of MD subjects refer of having at least 1 familiar with recurrent vertigo episodes.

In elderly MD subjects, we found a lower rate of positivity for autoantibodies, lifetime history of migraine and familiar history of recurrent vertigo.

These results and the presence of a higher rate of microischemic lesions are not inconsistent with the hypothesis of a different etiological mechanism, possibly related to vascular disorders, in the genesis of MD in elderly patients, and further studies should assess the question.

Elderly patients commonly present other vascular disorders, and these factors could influence the clinical features of MD. The presence of vascular problems in elderly MD patients has been found in other studies, and a higher prevalence of hypertension in MD patients has been reported in a population of 131 subjects [36]. Other studies have focused on a correlation between carotid atheromatosic changes and peripheral vestibular disorders, in some cases mimicking MD [37, 38].

A more recent work reported the presence of microischemic lesions of brain white matter in 31% of MD subjects, while only 25% of BPPV subjects, condition in which a vascular disorder has been demonstrated to act as a predisposing factor [39].

As a final consideration, MD in elderly patients may produce more disrupting results, since most of them present decreased postural and gait control due to a physiological reduction of the motor and sensorial system. Functional balance in elderly subjects with chronic vestibular disorders is worsened when associated with aging, concurrent disorders, use of multiple medications, central vestibular syndromes, mobility, and gait impairments.

## 5. Conclusions

Hydrops may be more related to a pathophysiological mechanism rather than a specific etiology. Our data support the hypothesis of different, possibly more related to a vascular disorder, predisposing factors for MD in elderly patients compared with MD in younger subjects.

Our data confirm previous works reporting a higher prevalence of Tumarkin attacks in elderly MD subjects; the lower rate of positive autoantibodies may be related to different etiopathological mechanisms of MD in elderly subjects.

Moreover, our data support the hypothesis of a more aggressive pattern in over-65 de novo occurrence MD patients.

## Figures and Tables

**Table 1:** Demographic and clinical data in elderly and younger MD subjects.

	Young MD (*n* = 73)	Elderly MD (*n* = 30)	*P* value
Age	50.1 ± 12	72 ± 4.3	*P *≤ 0.01
Age at onset of MD	39 ± 9.7	69.3 ± 4.1	*P* ≤0.01
Migraine	38/73 (51%)	4/30 (20%)	*χ* ^2^ = 8.93 *P * = 0.03
Positive autoantibody screening	25/73 (33.8%)	3/30 (20%)	*χ* ^2^ = 6.31 *P* = 0.02
Tumarkin	7/73 (9.6%)	11/30 (36-7%)	*χ* ^2^ = 10.8 *P* ≤0.01
Familiar history of vertigo	8/73 (10.9%)	2/30 (6%)	*χ* ^2^ = 0.5 *P* = 0.5
Number of spells in the last 6 months	5.5 ± 3.7	10.4 ± 5.4	*P* ≤ 0.05

**Table 2:** Values of hearing loss in affected and unaffected ear (mean value of frequencies between 250 and 2000 hz), number of vertigo spells in the last 6 months, and years since the first attack in the group of 30 elderly subjects and in the subgroup of 32 patients with a lower of 6 years history of vertigo.

	Elderly MD subjects (*n* = 30)	Younger MD subjects (*n* = 32)	*P* value
Years since first vertigo	2.7 ± 2.3	3.4 ± 1.8	0.08
Hearing loss affected ear	64.7 ± 8.1 db	48.2 ± 10.9 db	0.001
Hearing loss unaffected ear	18.2 ± 4.5 db	16.3 ± 3.3 db	0.04
N° of crises in the last 6 months	6.5 ± 3.7	10.4 ± 5.5	0.002
